# Optimization of the Switch Mechanism in a Circuit Breaker Using MBD Based Simulation

**DOI:** 10.1155/2015/347047

**Published:** 2015-03-30

**Authors:** Jin-Seok Jang, Chang-Gyu Yoon, Chi-Young Ryu, Hyun-Woo Kim, Byung-Tae Bae, Wan-Suk Yoo

**Affiliations:** ^1^School of Mechanical Engineering, Pusan National University, Busan 609-735, Republic of Korea; ^2^Agency for Defense Development, P.O. Box 126, Changwon, Gyeongnam 641-836, Republic of Korea; ^3^Hyosung Corporation, Changwon 641-712, Republic of Korea; ^4^Faculty of Mechanical Engineering, Pusan National University, Busan 609-735, Republic of Korea

## Abstract

A circuit breaker is widely used to protect electric power system from fault currents or system errors; in particular, the opening mechanism in a circuit breaker is important to protect current overflow in the electric system. In this paper, multibody dynamic model of a circuit breaker including switch mechanism was developed including the electromagnetic actuator system. Since the opening mechanism operates sequentially, optimization of the switch mechanism was carried out to improve the current breaking time. In the optimization process, design parameters were selected from length and shape of each latch, which changes pivot points of bearings to shorten the breaking time. To validate optimization results, computational results were compared to physical tests with a high speed camera. Opening time of the optimized mechanism was decreased by 2.3 ms, which was proved by experiments. Switch mechanism design process can be improved including contact-latch system by using this process.

## 1. Introduction

A circuit breaker is widely used to protect electric power system from fault currents or system errors. Spring actuated linkage system is a reliable mechanical device to transfer the stored elastic energy of the opening and closing spring to the mechanism composed of cams and links at a high speed. In particular, the opening mechanism is crucial in a circuit breaker to protect the electric systems under the emergency of current overflow.

Since the spring-type operation mechanism is composed of cams, several links, and springs, the system is rather complex [1]. Computer simulation such as multibody dynamic analysis had been widely used to analyze these kinds of multibody systems. For an advanced design of a circuit breaker, however, the designer has to estimate the accurate load history reacting on all the moving links and joints for various operation conditions. For this reason, a multibody dynamic analysis was necessary to estimate and validate dynamic characteristic and analyze the operating time. Ahn and Kim [2] applied the lumped parameter spring model in the vacuum circuit breaker to carry out the dynamic analysis of the circuit breaker. Pisano and Freudenstein [3] measured the dynamic performance of high speed cam-follower system by experiment. Yoo et al. [4] studied the spring actuated linkage system in circuit breaker system using MBD analysis program. Jang et al. [5] researched the possibility of cam profile optimization used in a spring actuated linkage system. To increase the stem velocity with the lowest spring force, many links are used in a circuit breaker. In the previous researches, dynamic analysis of the system was mainly focused.

In this paper, dynamic analysis technique was applied to design the circuit breaking mechanism. To start an optimal design, a precise multibody modeling of the circuit breaker was first developed. After the dynamics simulation with the developed multibody model, physical experiments were carried out to verify the simulation. Verifying the simulation results, an optimization process was adopted for the system to shorten the operating time. Since the switch mechanism has several contact conditions, such as roller-latch, solenoid plunger-latch, and latch stopper, design parameter is required to satisfy the contact condition to keep the closing condition. In this study, design parameters were selected from each length and shape of latches and bearing pivot points. Parametric study was first carried out and the optimization software VisualDOC was employed with dynamics analysis program MSC.ADAMS and electromagnetic actuator analysis software AMESim. To validate optimization results, an opening test was carried out with the optimum results. A high speed camera more than 3,000 frames per second was used to capture the motion and analyze the tracking points. For design of switch mechanism including contact conditions of several steps, switch mechanism design process can be improved.

This paper is structured as follows.  Section 2 explains the circuit breaker mechanism and the multibody system modeling including the solenoid. Construction of the coupled analysis system is explained. In Section 3, parameterization of switch parts is explained. And Section 4 shows the optimization procedure and validation results through the experiment using high speed camera. Finally, summary and conclusions are drawn in Section 5.

## 2. Dynamics Model

### 2.1. Multibody Dynamics Model

**Figure 1:**
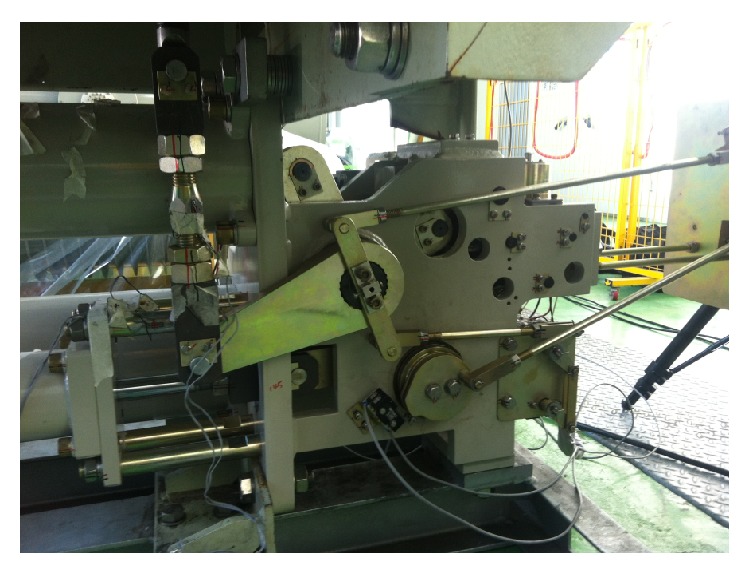
A circuit breaker.

**Figure 2:**
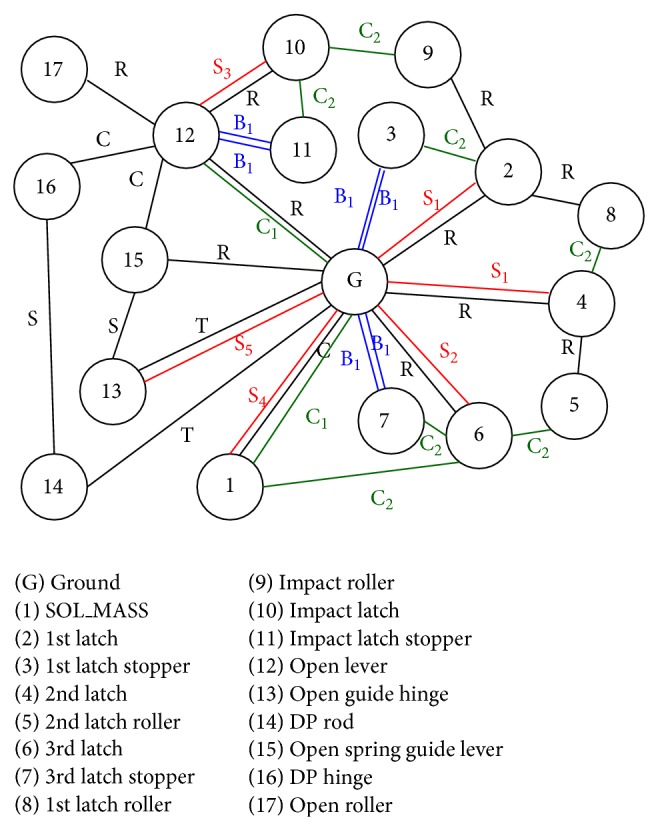
Graphical topology of a circuit breaker.

Figure 1 shows a circuit breaker model used in this research, and graphical topology map of the circuit break system is shown in Figure 2. Each number in circles means body and “S” means a spring element. Symbols “B,” “C,” “R,” “S,” and “T” mean bushing force element, contact force model, revolute joint, spherical joint, and translational joint, respectively. Total system consists of 18 bodies, and it has 29 degrees of freedom as shown in Table 1.

**Figure 3:**
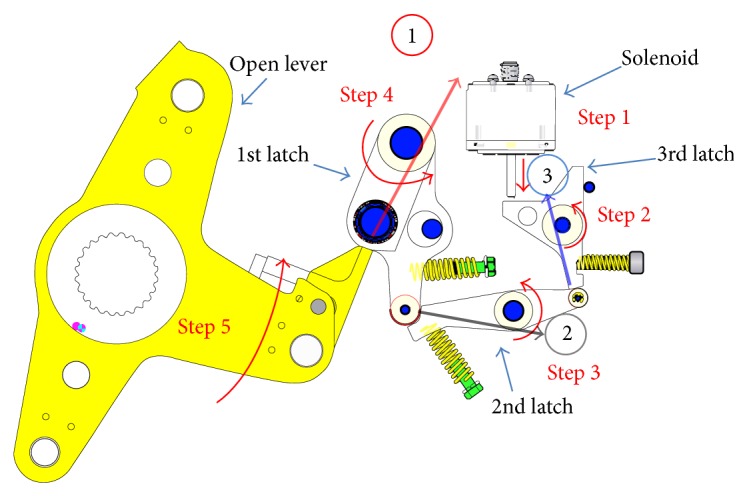
Operation sequence of switch parts.

Several latch stoppers are connected to ground by bushing element to represent flexible mount effects and vibration reduction. Therefore, many DOF appear in this switch mechanism, which have several latches, roller, stopper, and solenoid. Operation sequence of a switch mechanism in circuit breaker system is explained in Figure 3.

**Step 1 .**

When an emergency situation occurs in a circuit breaker system, the first movement occurs in the plunger of a solenoid.

**Step 2 .**

Plunger pushes the 3rd latch and then the 3rd latch starts to rotate counter clockwise. And then, contact between the 3rd latch and the 2nd latch is released.

**Step 3 .**

Releasing the contact with the 3rd latch, the 2nd latch rotates counterclockwise.

**Step 4 .**

Releasing the contact with the 2nd latch, the 1st latch rotates counterclockwise.

**Step 5 .**

Releasing the contact with the 1st latch, the open lever rotates counterclockwise. Then, the circuit is open, which means the circuit breaker is successfully done.

In this study, the operating time starting from Steps 1 to 5 was chosen as an objective value since circuit breaker finishes its role. In Figure 3, contact forces at a static equilibrium position were also drawn.

### 2.2. Analysis and Validation with the Developed Multibody Model

**Figure 4:**
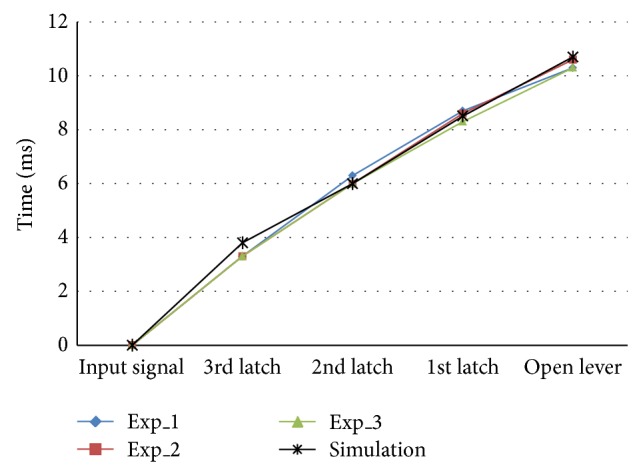
Comparison results between experiments and simulation.

Figure 4 shows comparison results between experiments and simulation. Each point in Figure 4 means time to move the latches and open lever. In particular, the time difference between experiments and simulation is within 0.4 ms. Since the discrepancy between experiment and simulation was small, it could be said that the multibody model was verified. Thus, optimization was carried out using this verified multibody dynamics model. 

## 3. Design Optimization

### 3.1. Selection of Design Variables

**Figure 5:**
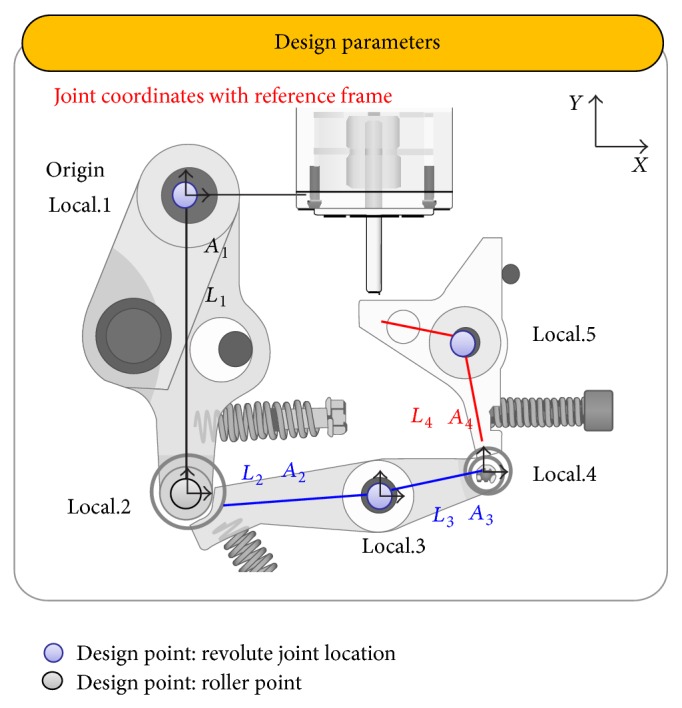
Selection of design parameters.

**Figure 6:**
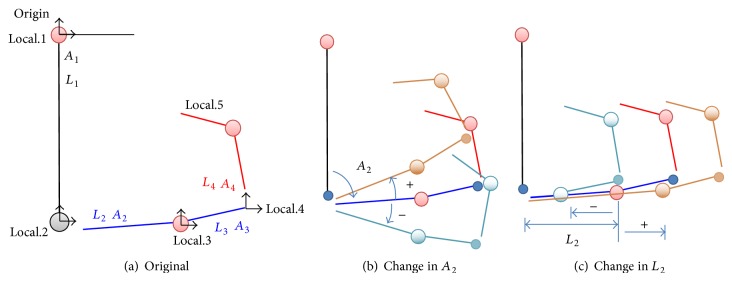
Configurations of switch mechanism according to parameter changes.

In Figure 5, selected design variables are shown, and their physical meaning was illustrated in Table 2. In switching part, roller point and pivot points of latches are selected as design parameters, in which positions and angles were measured from local reference frame. In Table 2, upper and lower limits of the design parameters were shown. These limits were considered by limitation of installation range and manufacturing conditions. Five of these parameters such as “*A*_2_,” “*A*_4_,” “*L*_2_,” “*L*_3_,” and “*L*_4_” were selected for parametric study, which were judged by an expert designer as main parameters. In the sequential operation from Steps 1 to 5, the contact condition should be preserved. Therefore these conditions were considered as boundary limits of design parameters shown in Table 2. Equation (1) explains global position of roller and pivot points which were named as Local.1, Local.2, Local.3, Local.4, and Local.5.  Figure 6 shows configurations of switch parts when the parameter changes “*A*_2_” and “*L*_2_,” representatively. If *A*_2_ and *L*_2_ change, global position of “local.3”, “local.4,” and “local.5” is calculated by using (1). The following method has many advantages, in checking static equilibrium condition for keeping the closing condition and in analyzing independent characteristics of switch part. Consider

(1) $$\begin{array}{c} \text{Local.1}=\left[\begin{array}{c} 0\\ 0 \end{array}\right]\\ \text{Local.2}=\text{Local.1}+\left[\begin{array}{c} L_{1}\sin\left(A_{1}\right)\\ {-L}_{1}\cos\left(A_{1}\right) \end{array}\right]\\ \text{Local.3}=\text{Local.2}+\left[\begin{array}{c} L_{2}\cos\left(A_{1}+A_{2}\right)\\ L_{2}\sin\left(A_{1}+A_{2}\right) \end{array}\right]\\ \text{Local.4}=\text{Local.3}+\left[\begin{array}{c} L_{3}\cos\left(A_{1}+A_{2}+A_{3}\right)\\ L_{3}\sin\left(A_{1}+A_{2}+A_{3}\right) \end{array}\right]\\ \text{Local.5}=\text{Local.4}+\left[\begin{array}{c} {-L}_{4}\sin\left(A_{1}+A_{2}+A_{3}+A_{4}\right)\\ L_{4}\cos\left(A_{1}+A_{2}+A_{3}+A_{4}\right) \end{array}\right]. \end{array}$$

### 3.2. Objective Function in Optimal Design

To shorten the total operation time for a circuit breaker, the total time for the five steps previously mentioned in Section 2.1 should be analyzed. Thus, object function was selected the time when the open lever is rotated 0.1 degree:

(2) $$\begin{array}{c} \text{Opening}\;\text{time}=\text{Opening}\;\text{lever}\;\text{angle}>0.1^{{}^{\circ}}. \end{array}$$

## 4. Optimization Results and Validation

### 4.1. Optimization Process

When a circuit breaker detects the fault current or system errors, the opening mechanism in the circuit breaker starts from the solenoid operation shown in Figure 5. Therefore solenoid model is important to develop a model for switch mechanism analysis.

**Figure 7:**
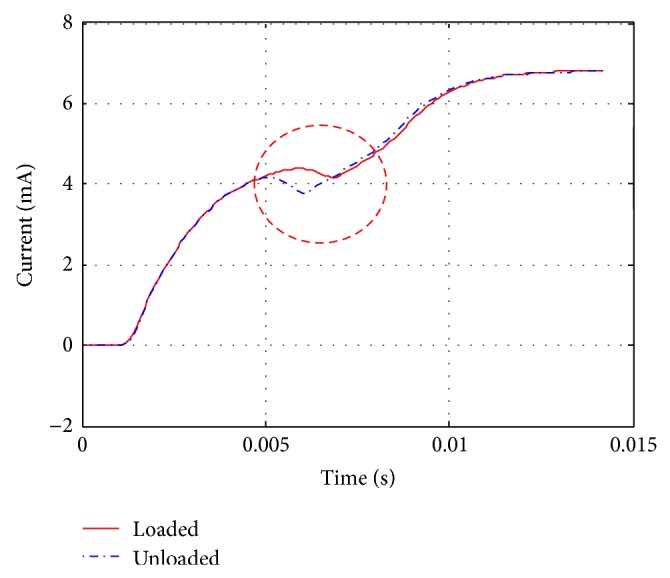
Current responses between loaded and unloaded condition.

**Figure 8:**
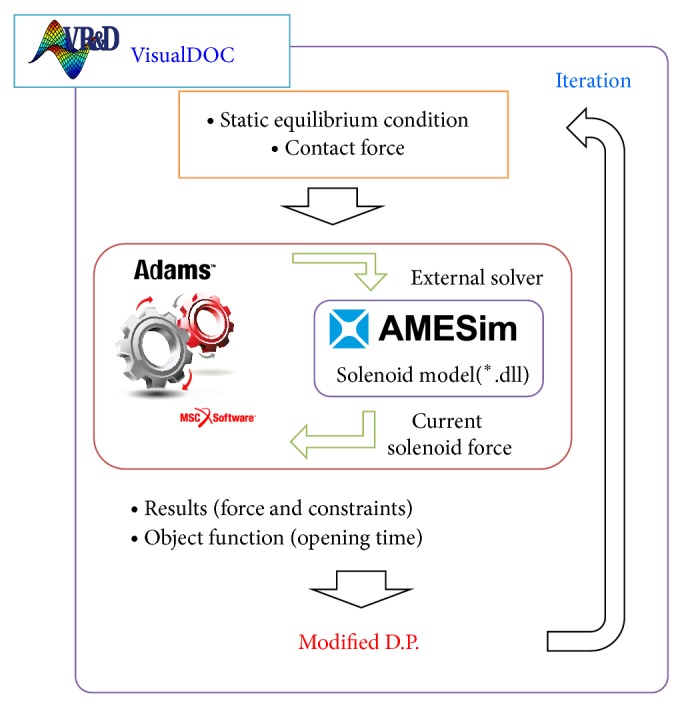
Optimization process of coupled system.

In this study, a multibody analysis code MSC.ADAMS called AMESim program as an external solver to analyze a fluid mechanical system and electromagnetic system. First, the solenoid model is developed in the AMESim and called by external solver in the MSC.ADAMS.  Figure 7 shows the comparison between unloaded case and loaded case applied by AMESim. Difference in current shows the necessity to consider the magnetic interaction in the solenoid because current applied to solenoid force is different.  Figure 8 shows the coupled system modeling based on MSC.ADAMS and optimization process on VisualDOC, in which VisualDOC program can integrate process and optimization [6, 7]. Static equilibrium conditions and contact force are estimated for satisfying the closing condition. Multibody dynamics analysis was carried out with MSC.ADAMS using the predeveloped solenoid model in external solver AMESim. Optimization was carried out using a genetic algorithm supplied in the VisualDOC. Genetic algorithm searches heuristic that mimics the process of natural evolution, and this heuristic is used to generate useful solutions to optimization. Algorithm condition was chosen for probability of crossover as 1.0, probability of mutation as 0.1, and population size as 100.

### 4.2. Optimization Results

**Figure 9:**
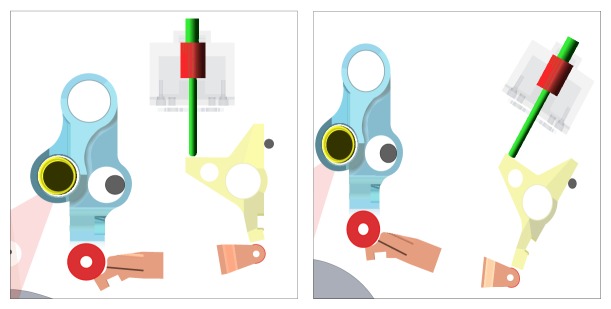
Geometry configuration shapes of existing model and optimal model.

Table 3 shows optimization results of the switch mechanism. In case of the original mechanism, opening time was 10.86 ms. After the optimal design, the opening time was reduced to 8.16 ms. Improvement of 24.8% was obtained with the time reduction of 2.7 ms.  Figure 9 shows the comparison between the original design and the advanced design after optimization. Table 3 shows optimization results of the switch mechanism. In case of the original mechanism, opening time was 10.86 ms. After the optimal design, the opening time was reduced to 8.16 ms. Improvement of 24.8% was obtained with the time reduction of 2.7 ms.

### 4.3. Experimental Validation

**Figure 10:**
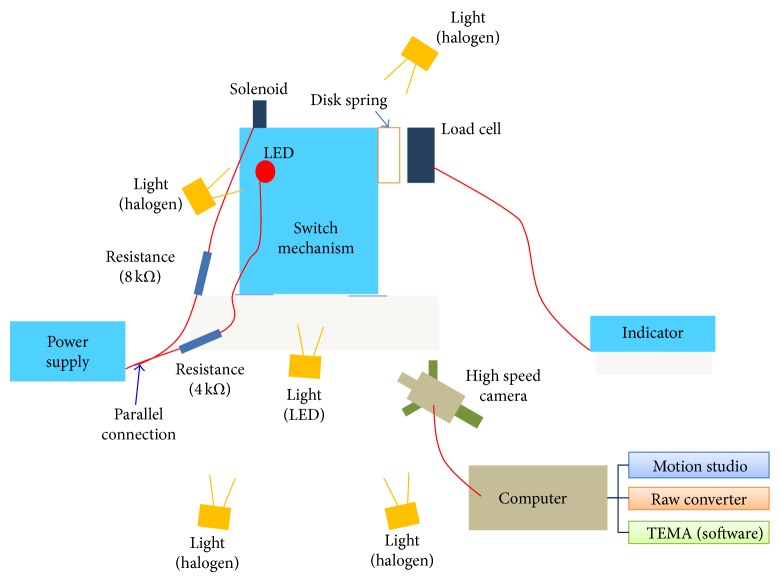
Diagram of experiments set for switch mechanism by using high speed camera.

**Figure 11:**
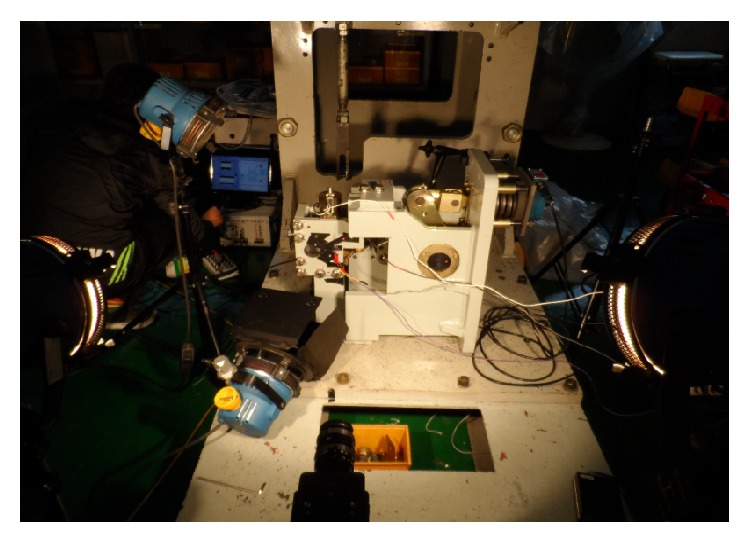
Picture of experimental setup.

To validate the optimization result, experiment of switch mechanism was also carried out with the design changes. Firstly housing and latches were newly manufactured and assembled. Since the opening process is finished with several milliseconds, therefore a high speed camera was used to capture the motion. Experiment setup is shown in Figures 10 and 11. Several lights were used to secure a clear view for high speed camera, and load cells and indicators were installed in order to conduct the same condition of spring force. During the motion capturing process, an LED lamp was used to check the start time. The latches motions were captured in 3000 frames per second, and then the captured data was converted to position data using TEMA software [8].

### 4.4. Validation Results

**Figure 12:**
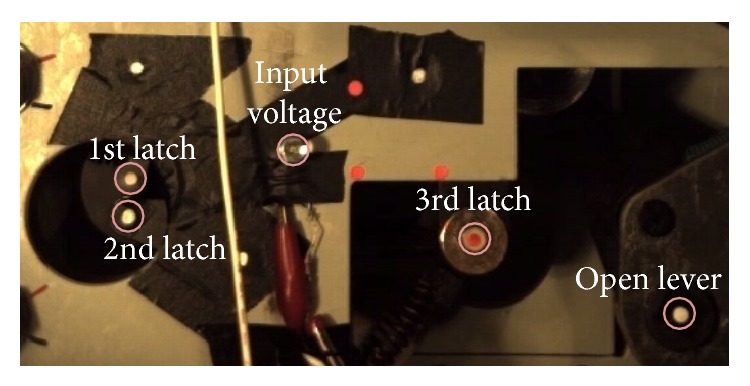
Markers in the experimental setup.

**Figure 13:**
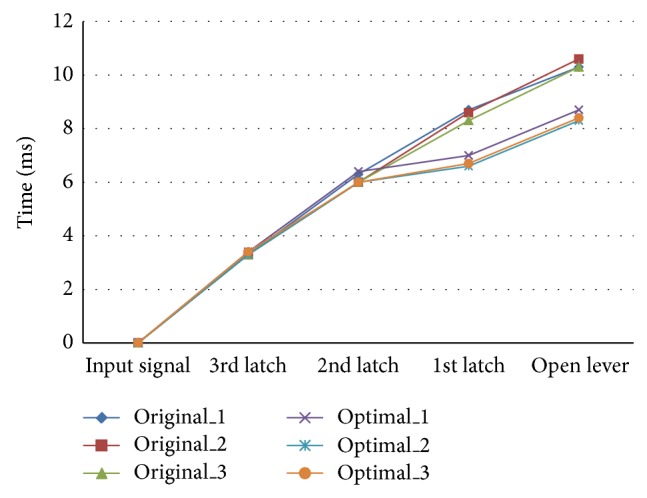
Times to move the latches and the open lever.

**Figure 14:**
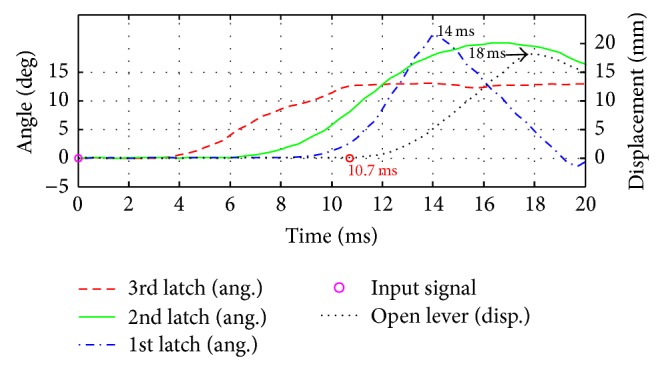
Displacement of latches and open lever (original mechanism).

**Figure 15:**
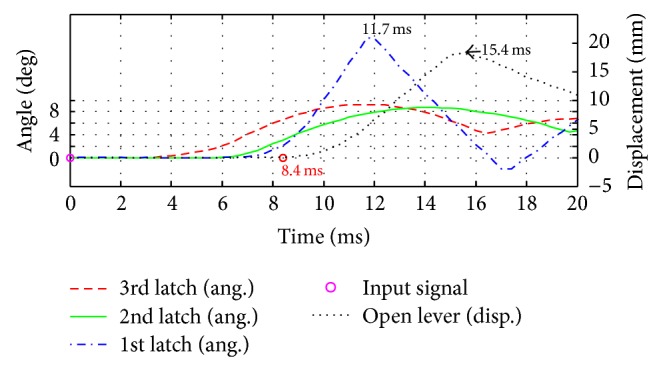
Displacement of latches and open lever (optimized mechanism).

To get the position data according to time, the points of each latch were tracked by markers, which were attached to latches and open lever as shown in Figure 12. Also an LED was also installed to check the start time as shown in Figure 12. To validate experiment reproducibility, experiments were carried out three times repeatedly. In Figure 13, measured times to move the open lever were shown, in which three curves with original model and other three curves with the optimized design were compared. Figures 14 and 15 compare displacement of latches and open lever with original design and the optimized design, respectively. By comparing two figures, optimally designed mechanism has faster operation time than the original mechanism. “Input signal” in figures means the time when the current was applied. As shown in figures, the 3rd latch and the 2nd latch start to rotate at 3.3 ms and 6 ms, respectively. After two latches rotating, in case of optimally designed mechanism, responses of the 1st latch are faster than the original mechanism about 2.0 ms. Start time to rotate the open lever was decreased by 2.3 ms. Since the start time is difficult to judge as shown in Figures 14 and 15, the time for the maximum open lever displacement was compared. Judging from the time, the opening time was decreased by 2.6 ms in experiments. Additionally, the rotation ranges of the 3rd and the 2nd latch in the optimally designed mechanism are smaller than those at original mechanism. However, open lever and the 1st latch have the same moving range.

## 5. Conclusion

Multibody dynamics model of a circuit breaker system was developed and the coupled analysis model was developed including electromagnetic actuator. Parameterization of design variable was carried out, and static equilibrium condition for the closing station was checked for the design variables. In the optimization process, the object function was defined to minimize the time for rotating of the open lever 0.1 degree.

Optimization was carried out using a genetic algorithm with the ViualDOC program. As a result, opening time was decreased by 2.7 ms in simulation. To verify the optimization results, experiments were also carried out with the optimal design components. For the experimental setup, a high speed camera was used to capture the motion with several indicators, load cells, lamps, and LED lamp. Three repetitive experiments were carried out for verifying the experimental reproducibility. The results showed that the opening time was decreased by 2.3 ms. Therefore, switch mechanism design process can be improved including contact-latch system by using this process.

## Figures and Tables

**Table 1:** DOF of the system.

Classification					DOF
Bodies	18	∗	6	=	108
Cylindrical joints	3	∗	−4	=	−12
Revolute joints	10	∗	−5	=	−50
Spherical joints	2	∗	−3	=	−6
Translational joints	2	∗	−5	=	−10
Motion	1	∗	−1	=	−1

Total DOF					29

**Table 2:** Design parameters and boundary values.

	Description	Lower limits	Upper limits	Selection criteria for boundary values
*A*_1_	Angle (deg)	−10	10	Interruption of parts
*A*_2_	Angle (deg)	−**30**	**10**	Closing condition
*A*_3_	Angle (deg)	−15	15	Interruption of parts
*A*_4_	Angle (deg)	−**30**	**10**	Closing condition
*L*_1_	Length (mm)	−3	3	
*L*_2_	Length (mm)	−**10**	**15**	Effects on the opening time and minimum length
*L*_3_	Length (mm)	−**4**	**4**	
*L*_4_	Length (mm)	−**7**	**10**	

**Table:** (a)

	Initial value	Optimum value
*L*_2_ (mm)	0	15.0
*L*_3_ (mm)	0	−3.5
*L*_4_ (mm)	0	5.0
*A*_2_ (deg)	0	−28.5
*A*_4_ (deg)	0	−28.5

**Table:** (b)

Optimization	
Opening time of existing mechanism	10.86 ms
Opening time of optimal mechanism	8.16 ms
Reduced time	2.7 ms
Improvement (%)	24.8
Total computing time	3.2 hours
